# Relationships of Serum Bone Turnover Markers With Metabolic Syndrome Components and Carotid Atherosclerosis in Patients With Type 2 Diabetes Mellitus

**DOI:** 10.3389/fcvm.2022.824561

**Published:** 2022-04-25

**Authors:** Wei Li, Xiaojun Liu, Lijun Liu, Lei Zhang, Mengdi Li, Rui Liu, Tianfang Li, En Chen, Shengyun Liu

**Affiliations:** ^1^Department of Rheumatology, The First Affiliated Hospital of Zhengzhou University, Zhengzhou, China; ^2^Department of Laboratory Medicine, The First Affiliated Hospital, Hengyang Medical School, University of South China, Hengyang, China

**Keywords:** bone turnover biomarkers, metabolic syndrome, carotid artery plaques, carotid atherosclerosis, type 2 diabetes mellitus

## Abstract

**Objective:**

This study aimed to investigate the association of serum bone turnover markers (BTMs) with metabolic syndrome components and carotid atherosclerosis in patients with type 2 diabetes mellitus (T2DM).

**Methods:**

We performed a cross-sectional based study in T2DM populations. Serum BTMs including N-terminal osteocalcin (N-MID), β-cross-linked C-telopeptide of type I collagen (β-CTX), and procollagen type I N-terminal propeptide (PINP) were measured by immunoassay method. Carotid artery intima-media thickness and carotid artery plaque (CAP) were measured by B-mode ultrasound.

**Results:**

The serum N-MID, PINP, and β-CTX levels significantly lower in the CAP group compared with the non-CAP group. N-MID and PINP levels were inversely associated with fasting blood glucose, HOMA-IR, CRP, eGFR, and triglycerides (all P < 0.05), whereas β-CTX levels were negatively associated with triglycerides (P < 0.05). After multiple adjustment, the odds ratios (ORs) were substantially higher for CAP with decreased N-MID level (OR = 0.958; 95% CI = 0.926–0.991; P = 0.013). However, serum levels of PINP and β-CTX were not associated with the presence of CAP. Multivariate logistic regression analysis further revealed that serum N-MID, PINP, and β-CTX levels were significantly associated with hypertriglyceridemia, whereas serum N-MID and β-CTX levels were associated with overweight/obesity risk.

**Conclusions:**

These findings indicated that serum N-MID level was an independent risk factor for carotid atherosclerosis, whereas BTM levels were associated with other metabolic syndrome components in a T2DM population.

## Background

Bone turnover is a dynamic process that comprises the formation of new bone by osteoblasts and the resorption of old bone by osteoclasts. This process generates many bone turnover markers (BTMs) from bone cells and the bone matrix, which provide a noninvasive evaluation of bone remodeling status (1, 2). BTMs comprises bone formation markers, namely, N-terminal osteocalcin (N-MID), procollagen type I N-terminal propeptide (PINP), and bone resorption markers, such as β-cross-linked C-telopeptide of type I collagen (β-CTX). All these molecules are key markers of bone metabolism (3, 4). In addition to the use of BTMs as indicators of bone formation and resorption, as well as markers for the assessment, diagnosis, and treatment of osteoporosis, BTMs have been shown to be associated with energy metabolism in recent studies (5, 6).

Previous studies have shown that bone metabolism is affected by the risk factors of atherosclerosis, such as obesity, hypertension, dyslipidemia, and diabetes (7–9). Patients with diabetes are at two-to fourfold increased risk of cardiovascular disease (CVD), which is reflected by atherosclerosis (10). BTMs are highly associated with diabetes, insulin sensitivity, and beta cell function, and serum osteocalcin plays an important role in the association of bone metabolism with glucose metabolism (11, 12). A cross-sectional based study has demonstrated that serum osteocalcin level is an independent risk factor for carotid atherosclerosis in patients with T2DM (13). In addition, previous observational and indirect interventional studies suggested a relationship between serum osteocalcin level with metabolic homeostasis and CVD (14). However, the relationship of other BTMs with metabolic syndrome and carotid atherosclerosis risk is substantially unknown. Recently, a case-control study has shown that higher osteopontin concentrations are associated with increased CVD risk in T2DM patients, but no association is found for the osteocalcin level and risk of CVD (15). Furthermore, a longitudinal follow-up study demonstrated that the serum total osteocalcin level was not associated with the development of CVD after adjusting for other risk factors (16).

Clinical studies have revealed that the association between different BTMs and cardiometabolic risk is controversial, and most studies have focused on osteocalcin (15, 16). Accordingly, the present study aimed to determine whether serum N-MID levels and other BTMs were independently associated with carotid atherosclerosis and other cardiometabolic risk factors, such as obesity, hypertension, dyslipidemia, and metabolic syndrome (MetS) in patients with T2DM.

## Methods

### Study Populations

Cross-sectional study populations were recruited from the First Affiliated Hospital of Zhengzhou University between 2018 and 2020. Diabetes was defined as fasting blood glucose ≥7.0 mmol/l, HbA1c≥ 6.5% or the use of any antidiabetic medication or self-reported history of diabetes based on the American Diabetes Association. Subjects were excluded if they had any known infection, malignant tumors, or were taking medicine that may influence the level of serum BTMs. Finally, a total of 1520 patients with T2DM were enrolled. Questionnaires were used to identify history of medical conditions, family history of disease, current medication use, and other lifestyle factors. Body weight and height were measured at baseline, and body mass index (BMI) was calculated by body weight (kg) divided by height square (m^2^). Blood pressure was measured using an automatic blood-pressure meter after seating for at least 10 min. The average of three measurements was recorded for further analysis. This study was approved by the Institutional Review Broad of the First Affiliated Hospital of Zhengzhou University.

### Biochemical Measurements

Venous blood samples were collected in the morning followed by overnight fasting. An auto-biochemical analyzer was used to determine the fasting blood glucose (FBG), serum concentrations of total cholesterol (TC), triglycerides (TG), low-density lipoprotein cholesterol (LDL-C), high-density lipoprotein cholesterol (HDL-C), uric acid (UA), fasting plasma insulin, creatinine, and C-reactive protein (CRP) as previously described. Glycated hemoglobin (HbA1c) concentrations were quantified using high-performance liquid chromatography. The estimated glomerular filtration rate (eGFR) was calculated according to the CKD-EPI equation. Insulin resistance was estimated using homeostasis model assessment index-insulin resistance (HOMA-IR). BTMs associated with bone metabolism including N-MID, PINP, and β-CTX were measured by electrical chemiluminescent immunoassay.

### Carotid Ultrasonography

Trained technicians performed B mode ultrasonography using an Acuson Sequoia. A lateral view of bilateral images of common carotid arteries (1 cm proximal to the dilatation of the carotid bulb), carotid bulb, and internal carotid artery was obtained. Carotid artery intima-media thickness (CIMT) was defined as the mean of the maximum thickness in both right and left sides of the common carotid artery, and IMT is the distance between the lumen-intima interface and the media adventitia interface. The carotid artery plaque (CAP) was defined as either a focal structure that encroaches into the arterial lumen by at least 50% of the surrounding IMT value or a thickness of > 1.5 mm. CAP presence was defined as ≥1 plaque in any of the carotid arteries.

### Definition of MetS

We used the definition of MetS according to the NCEPATP III criteria (17). Subjects were classified as having MetS when ≥3 of the following criteria were present: FBG level ≥5.6 mmol/L, blood pressure ≥130/85 mmHg, TG ≥1.7 mmol/L, HDL-C < 1.03 mmol/L for men and < 1.29 mmol/L for women, and waist circumference >102 cm for men and >88 cm for women.

### Statistical Analysis

Normally distributed data were expressed as the mean ±SD, whereas variables with a skewed distribution were reported as median (interquartile range). Categorical variables were represented by percentage. Mann–Whitney *U-*test was used to compare the mean ranks between the CAP and non-CAP groups. Correlation coefficients between BTMs and metabolic features were calculated by partial correlation analysis. Multivariate logistic regression models were used to estimate the association of CAP with BTMs. Potential confounding variables including age, gender, smoking, alcohol drinking, self-reported CVD, hypertension, CRP, BMI, FBG, HbA1c, HOMA-IR, TG, TC, HDL-C, and LDL-C were controlled in the regression models. Statistical analyses were performed using SPSS version 26.0 (Chicago, IL, USA). Results were considered statistically significant at *P* < 0.05.

## Results

### Study Population and Characteristics

The baseline clinical characteristics of the participants are shown in Table 1. A total of 1520 subjects were enrolled in the present study, among which 695 (45.7%) had CAP. Participants were classified according to the presence of carotid plaques as CAP and non-CAP groups. The CAP group had significantly lower systolic blood pressure (SBP), diastolic blood pressure (DBP), BMI, TC, TG, and UA levels, as well as eGFR, HOMA-IR, this group also had a higher frequency of hypertension and self-reported CVD (all *P* < 0.05). The CAP group had higher HDL-C levels and longer diabetes duration. Furthermore, serum N-MID, PINP, and β-CTX levels significantly lower in the CAP group compared with the non-CAP group (all *P* < 0.05).

**Table 1:** Baseline characteristics for participants.

	**Non-CAP**	**CAP**	***P*-value**
*N* (%)	825 (54.3%)	695 (45.7%)	
Male, *n* (%)	535 (64.8%)	466 (67.1%)	**<0.0001**
Age (years)	45 (34–54)	56 (48–62)	**<0.0001**
Diabetes duration (years)	2 (0.17–6)	5 (1–12)	**<0.0001**
History of CVD, *n* (%)	46 (5.6%)	93 (13.4%)	**<0.0001**
Hypertension, *n* (%)	293 (35.5%)	368 (52.9%)	**<0.0001**
Smoking, *n* (%)	199 (24.1%)	185 (26.6%)	0.624
Drinking, *n* (%)	162 (19.6%)	161 (23.2%)	0.094
BMI (kg/m^2^)	26.2 (23.7–29.9)	25 (23–27)	**<0.0001**
CRP (mg/L)	1.29 (0.62–3.26)	1.22 (0.56–2.64)	0.157
HbA1C (%)	8.3 (6.7–10.4)	8.5 (7.2, 10.0)	0.858
FBG (mmol/L)	7.3 (6.0–10.3)	7.6 (6.2–10.0)	0.84
Insulin (μU/mL)	5.9 (2.8–10.5)	5.0 (2.3–9.4)	**0.019**
HOMA-IR	2.06 (1.01–3.47)	1.70 (0.77–3.14)	**0.015**
UA (μmol/L)	304 (247–378)	286 (247–347)	**0.023**
eGFR	111.1 (101.5–120.4)	102.9 (94.3–109.9)	**<0.0001**
TC (mmol/L)	4.54 (3.9–5.3)	4.45 (3.7–5.2)	**0.003**
TG (mmol/L)	1.9 (1.1–3.1)	1.8 (1.2–2.5)	**<0.0001**
HDL-C (mmol/L)	1.01 (0.81–1.24)	1.05 (0.89–1.25)	0.07
LDL-C (mmol/L)	2.75 (2.12–3.36)	2.65 (1.94–3.24)	0.058
N-MID (ng/mL)	12.6 (10.4–16.0)	11.8 (9.1–15.5)	**0.019**
β-CTX (ng/mL)	0.4 (0.29–0.55)	0.35 (0.25–0.51)	**0.001**
PINP (ng/mL)	37.1 (29.3–49.9)	35.1 (25.3–45.4)	**0.021**
SBP (mmHg)	133 (125–141)	132 (124–145)	**0.004**
DBP (mmHg)	84 (77–90)	83 (76–90)	**0.004**
Antidiabetic, *n* (%)	201 (24.4%)	279 (40.1%)	**<0.0001**
Antihypertensive, *n*(%)	501 (60.7%)	505 (72.7%)	**<0.0001**
Lipid lowering, *n* (%)	45 (5.5%)	83 (11.9%)	**<0.0001**

*β-CTX, β-cross-linked C-telopeptide of type I collagen; BMI, Body mass index; CAP, Carotid artery plaques; CVD, Cardiovascular disease; CRP, C-reactive protein; DBP, Diastolic blood pressure; eGFR, Estimated glomerular filtration rate; FBG, Fasting plasma glucose; HbA1c, Glycosylated hemoglobin; HDL-C, High-density lipoprotein cholesterol; LDL-C, Low-density lipoprotein cholesterol; N-MID, N-terminal osteocalcin; PINP, procollagen type I N-terminal propeptide; SBP, Systolic blood pressure; TC, Total cholesterol; TG, Triglyceride. The bold values indicated that difference is statistically significant*.

### Correlation Analysis of the Relationship Between BTMs and Biochemical Parameters

Spearman analysis showed that serum N-MID level was correlated with CRP (*r* = −0.127; *P* = 0.009), FBG (*r* = −0.235; *P* < 0.001), HbA1C (*r* = −0.224; *P* < 0.001), HDL-C (*r* = 0.105; *P* = 0.033), TG (*r* = −0.1; *P* = 0.041), eGFR (*r* = −0.19; *P* < 0.001), and SBP (*r* = 0.134; *P* = 0.006) after adjusting for age and sex. However, serum β-CTX was associated only with TG (*r* = −0.105; *P* = 0.032) and was not significantly associated with other parameters. Furthermore, serum PINP level was correlated with BMI (*r* = 0.149; *P* = 0.002), CRP (*r* = −0.103; *P* = 0.035), FBG (*r* = −0.233; *P* < 0.001), HbA1C (*r* = −0.187; *P* < 0.001), fasting plasma insulin (*r* = 0.193; *P* < 0.001), HOMA-IR (*r* = 0.098; *P* = 0.045), eGFR (*r* = −0.162; *P* = 0.001), and TG (*r* = −0.13; *P* = 0.007) after adjusting for age and sex (Table 2). However, the serum BTM levels was not independently associated with CIMT in all study populations.

**Table 2:** Correlation between BTMs and other parameters in patients with T2DM.

**Variables**	**N-MID**		**β-CTX**		**PINP**	
	**r**	***P*-value**	***r***	***P*-value**	***r***	***P*-value**
BMI	−0.002	0.967	0.047	0.333	0.149	**0.002**
CRP	−0.127	**0.009**	−0.042	0.392	−0.103	**0.035**
HbA1C	−0.224	**< 0.0001**	−0.026	0.598	−0.187	**<0.0001**
FBG	−0.235	**<0.0001**	−0.089	0.069	−0.233	**<0.0001**
HOMA-IR	−0.011	0.819	0.016	0.742	0.098	**0.045**
Insulin	0.071	0.15	0.029	0.559	0.193	**<0.0001**
eGFR	−0.19	**<0.0001**	−0.067	0.17	−0.162	**0.001**
UA	−0.006	0.903	−0.041	0.397	0.011	0.824
TC	0.021	0.676	−0.011	0.815	−0.031	0.52
TG	−0.1	**0.041**	−0.105	**0.032**	−0.13	**0.007**
HDL-C	0.105	**0.033**	0.066	0.179	0.051	0.296
LDL-C	0.075	0.124	0.05	0.306	0.056	0.255
SBP	0.134	0.006	0.02	0.689	0.042	0.385
DBP	0.08	0.105	−0.008	0.867	0.018	0.719
CIMT	0.084	0.286	0.118	0.131	0.06	0.452

*All correlation coefficients were calculated after adjustment for age, gender. The bold values indicated that difference is statistically significant*.

### Association Between Serum BTM Levels and CAP

Given that the BTM levels were significantly in inverse association with CRP, we adjusted for CRP in the multivariable logistic regression analysis. Table 3 shows that after adjusting for age, sex, BMI, and CRP, only reduced serum N-MID level were revealed to be significantly associated with increased risk of CAP (odds ratios (OR) 0.957; 95% CI = 0.927–0.988; *P* = 0.006). We obtained similar associations when the multivariable logistic regression analysis was further adjusted for smoking, alcohol drinking, duration of diabetes, hypertension, and history of CVD (OR = 0.958; 95% CI = 0.928–0.989; *P* = 0.008). Low serum N-MID indicated a high risk for CAP (OR = 0.958; 95% CI = 0.926–0.991; *P* = 0.013) after further adjusting for FBG, HbA1C, serum TC, TG, HDL-C, and LDL-C. However, the PINP and β-CTX levels were not significantly associated with the risk of CAP in all multivariable logistic regression models.

**Table 3:** Association of serum BTMs with CAP in T2DM populations.

	**OR (95% CI)**	***P*-value**
N-MID		
Model 1	0.957 (0.927–0.988)	**0.006**
Model 2	0.958 (0.928–0.989)	**0.008**
Model 3	0.958 (0.926–0.991)	**0.013**
β-CTX		
Model 1	0.631 (0.313–1.273)	0.198
Model 2	0.686 (0.334–1.407)	0.304
Model 3	0.672 (0.315–1.435)	0.304
PINP		
Model 1	0.995 (0.987–1.004)	0.283
Model 2	0.995 (0.987–1.004)	0.281
Model 3	0.995 (0.986–1.004)	0.287

*Model 1 adjusted for age, gender, BMI and CRP*.

*Model 2 further adjusted for alcohol drinking, smoking, duration of diabetes, hypertension, and history of CVD*.

*Model 3 further adjusted for FBG, HbA1C, TG, TC, HDL-C, and LDL-C*.

*The bold values indicated that difference is statistically significant*.

### Association Between Serum BTMs and MetS Components

Among MetS components, after adjusting for age, sex, BMI, CRP, smoking, alcohol drinking, duration of diabetes, hypertension, and history of CVD (model 2), overweight/obesity (BMI ≥ 25 kg/m^2^) were found to be negatively associated with N-MID (OR = 0.96; 95% CI = 0.94–0.99; *P* = 0.015) and β-CTX (OR = 0.446; 95% CI = 0.24–0.85; *P* = 0.014). Similarly, hypertriglyceridemia was negatively associated with N-MID (OR = 0.96; 95% CI = 0.93–0.99; *P* = 0.004) and β-CTX (OR = 0.34; 95% CI = 0.18–0.67; *P* = 0.002). Significant associations were also observed between the presence of hypertriglyceridemia and PINP after adjusting for potential confounders (OR = 0.99; 95% CI = 0.98–1.00; *P* = 0.003; model 2). However, no significant association was found between serum BTMs and the presence of MetS, hypertension, and HDL-C dyslipidemia in T2DM populations (Table 4).

**Table 4:** Association of serum BTMs with metabolic syndrome components.

**Variables**	**N-MID**		**β-CTX**		**PINP**	
	**OR (95% CI)**	***P-*value**	**OR (95% CI)**	***P*-value**	**OR (95% CI)**	***P*-value**
MetS	0.99 (0.95–1.03)	0.601	0.54 (0.24–1.21)	0.135	0.99 (0.98–1.00)	0.195
Overweight/Obesity	0.96 (0.94–0.99)	**0.015**	0.45 (0.24–0.85)	**0.014**	1.00 (0.99–1.01)	0.651
Hypertension	1.01 (0.98–1.04)	0.369	1.22 (0.63–2.34)	0.555	1.00 (0.99–1.01)	0.896
Dyslipidemia (TG)	0.96 (0.93–0.99)	**0.004**	0.34 (0.18–0.67)	**0.002**	0.99 (0.98–1.00)	**0.003**
Dyslipidemia (HDL-C)	1.00 (0.97–1.03)	0.736	0.85 (0.44–1.66)	0.637	1.00 (0.99–1.01)	0.708

*All variables adjusted for age-gender, BMI, CRP, alcohol drinking, smoking, duration of diabetes, hypertension, and history of CVD. The bold values indicated that difference is statistically significant*.

## Discussion

In the present study, we found an association between serum N-MID and the risk of carotid atherosclerosis in T2DM populations. These associations were independent of lifestyle factors, duration of diabetes, history of CVD, CRP, HbA1c, FBG, lipid parameters, and BMI. Multivariate logistic regression analysis revealed that serum N-MID, PINP, and β-CTX levels were significantly associated with hypertriglyceridemia, whereas serum N-MID and β-CTX levels were associated with overweight/obesity risk. These findings indicated the association between serum N-MID and metabolic syndrome components, as well as carotid atherosclerosis, independent of other known CVD risk factors in patients with T2DM, thus suggesting that BTMs (especially N-MID) were important for glucose and lipid metabolism, and atherosclerosis.

Several studies have shown that serum BTMs may be a predictor of CVD risk in patients with T2DM (15, 18). In a previous study, serum osteocalcin was negatively associated with parameters of atherosclerosis in T2DM patients (19). In another study, serum levels of osteocalcin were inversely associated with the metabolic syndrome and the severity of coronary artery disease in Chinese populations (20). Interestingly, consistent with previous studies, we observed that serum N-MID levels were an independent risk factor for carotid atherosclerosis in patients with T2DM. Endothelial dysfunction is considered as an early step in atherosclerosis development as it contributes to the initiation and early progression of atherosclerosis (21, 22). Some studies suggested that high serum osteocalcin level contributes to vascular calcification and atherosclerosis. However, different population lead to inconclusive results (23). Large longitudinal studies are needed to further explore the clinical relevance of serum osteocalcin in vascular calcification and atherosclerosis (24). In a mouse model of atherosclerosis, daily injections of osteocalcin reduced the risk of CVD, which showed an endothelial-protective effect because it improves glucose and lipid metabolism by activating the PI3K-Akt-eNOS signaling pathway (25). However, prospective studies are needed to clarify whether low osteocalcin level plays a causal role in atherosclerosis development.

The current study revealed that another bone formation marker, PINP level, was significantly associated with HOMA-IR, FBG, fasting insulin levels, CRP, and TG. These associations were reported in a previous study in which PINP was positively correlated with insulin sensitivity and negatively correlated with glucose and triglycerides (26). Significant associations were also observed between BTM levels and the presence of hypertriglyceridemia after adjustment for potential confounders. However, we did not find an association of serum PINP and β-CTX levels with CAP risk in T2DM populations. In view of the high correlation between PINP, β-CTX, and N-MID, the effects of PINP and β-CTX on carotid atherosclerosis were most likely to be attributable to the function of N-MID (11). Thus, the interactions among different BTMs require further investigations.

Vascular endothelial inflammation was considered to play a vital role in the mechanism of CVD development (27, 28). CRP is an acute-phase reactant and a well-known serum marker of chronic low-grade inflammation. It is associated with diabetes, hypertension, obesity, and CVDs (29, 30). In the present study, serum N-MID and PINP levels were negatively correlated with CRP. Accordingly, the results of current study may be partially attributed to the mechanism of chronic low-grade inflammation. Furthermore, elevated serum TG was an independent risk factor for CVD development (31). Consistent with previous studies, serum N-MID and PINP levels were negatively correlated with TG in our study (32). Meanwhile, after adjustment for potential confounders, all BMTs (including N-MID, β-CTX, and PINP) were negatively associated with hypertriglyceridemia. Therefore, given that serum N-MID levels were significantly associated with CRP and TG levels and the strong relationship of serum N-MID with carotid atherosclerosis risk, N-MID can be considered as a promising candidate for risk assessment and a potential intervention target for CVD.

A previous study has shown no significant correlation between CIMT and serum osteocalcin (33). In accordance with these findings, the present study demonstrated that the three BTMs were not significantly associated with CIMT in T2DM patients. Furthermore, clinical studies investigating the association of serum osteocalcin and CVD risk are controversial, and the lack of consistency may be due to different study populations or different degrees of confounding factors associated with serum osteocalcin level, such as metabolic factors and chronic low-grade inflammation, and these metabolic dysfunctions are related to the progression of atherosclerosis (34–36). In humans, bone turnover rate varies obviously according to individual variables, age and sex are the most important variables determining bone remodeling. Given that the serum levels of osteocalcin differ between sexes and alter with age, the relationship of serum osteocalcin levels with CVD risk may also differ according to these variables (37). Studies have demonstrated that close correlations of serum osteocalcin level with glucose and lipid metabolic disorders, obesity, and MetS (8, 38). Herein, the serum N-MID level was correlated with CRP, FBG, HbA1C, HDL-C, and TG after adjusting for age and sex. Similar associations were found in PINP, indicating that serum BTMs may play a key role in glucose and lipid metabolism. Thus, given the strong association between metabolic risk and atherosclerosis, our findings suggested that the association of BTMs with atherosclerosis may be influenced by metabolic variables.

## Conclusion

Our cross-section study suggested that serum N-MID levels were significantly associated with carotid atherosclerosis in patients with T2DM even after adjusting for potential confounders. Serum BTMs level were associated with other metabolic syndrome components, which may reflect the role of BTMs as a circulating endocrine markers regulating glucose and lipid metabolism, thereby posing a cardiovascular risk to T2DM patients.

## Data Availability Statement

The original contributions presented in the study are included in the article/supplementary material, further inquiries can be directed to the corresponding author/s.

## Ethics Statement

The studies involving human participants were reviewed and approved by Ethical Committee of the First Affiliated Hospital of Zhengzhou University. The Ethics Committee waived the requirement of written informed consent for participation.

## Author Contributions

WL, EC, XL, LZ, LL, TL, and SL contributed to the conception and design of the study. WL, SL, ML, and RL recruited the subjects and supervised the study. WL, EC, LZ, LL, and XL analyzed the data. WL, EC, and SL wrote the initial draft of the article. WL, EC, LZ, TL, XL, and SL contributed to the writing, reviewing, and revising of the manuscript. All authors contributed to the article and approved the submitted version.

## Funding

WL is funded by the National Natural Science Foundation of China (82000831).

## Conflict of Interest

The authors declare that the research was conducted in the absence of any commercial or financial relationships that could be construed as a potential conflict of interest.

## Publisher's Note

All claims expressed in this article are solely those of the authors and do not necessarily represent those of their affiliated organizations, or those of the publisher, the editors and the reviewers. Any product that may be evaluated in this article, or claim that may be made by its manufacturer, is not guaranteed or endorsed by the publisher.

## Abbreviations

β-CTX: β-cross-linked C-telopeptide of type I collagen
BMI: Body mass index
BTM: bone turnover markers
CAP: Carotid artery plaque
CI: Confidence interval
CIMT: Carotid intima-media thickness
CVD: Cardiovascular disease
CRP: C-reactive protein
DBP: Diastolic blood pressure
eGFR: Estimated glomerular filtration rate
FBG: Fasting plasma glucose
HbA1c: Glycosylated hemoglobin
HDL-C: High-density lipoprotein cholesterol
LDL-C: Low-density lipoprotein cholesterol
MetS: metabolic syndrome
N-MID: N-terminal osteocalcin
OR: Odds ratio
PINP: procollagen type I N-terminal propeptide
SBP: Systolic blood pressure
TC: Total cholesterol
TG: Triglyceride
T2DM: Type 2 diabetes mellitus
SUA: Serum uric acid.
